# The complete chloroplast genome sequence of *Malus toringo* (Rosaceae)

**DOI:** 10.1080/23802359.2020.1790317

**Published:** 2020-07-15

**Authors:** Yanan Li, Yanlei Liu, Chao Xu, Fengyi Li, Ling Wang, Shiliang Zhou

**Affiliations:** a College of Landscape Architecture, Northeast Forestry University, Harbin, China; b State Key Laboratory of Systematic and Evolutionary Botany, Institute of Botany, Chinese Academy of Sciences, Beijing, China; c College of Life Sciences, University of Chinese Academy of Sciences, Beijing, China

**Keywords:** *Malus toringo*, complete chloroplast genome, medicinal value, phylogenetic relationships

## Abstract

*Malus toringo* (Siebold) Siebold ex de Vriese is a worthy species in the genus Malus (family Rosaceae) distributing from North America and Asia, especially in China. In addition to its ornamental value, the medicinal value of its fruits has also been developed to treat food stagnation diseases. Herein, we obtained the complete chloroplast genome of *M. toringo* using next-generation sequencing technology. The complete chloroplast genome is 160,039 base pair (bp) in length, including a large single copy (LSC) region of 88,142 bp, a small single copy (SSC) region of 19,183 bp, and a pair of inverted repeat regions (IRs, 26,357 bp). And, the overall GC content of the chloroplast genome was 36.6%. Besides, a total of 128 unique genes were found in the chloroplast genome, namely 83 protein-coding genes (PCGs), 37 tRNA genes (tRNA), and eight rRNA genes (rRNA). A maximum-likelihood phylogenetic tree was reconstructed using the full length of chloroplast genome to show the phylogenetic relationships among species in genus Malus. It was concluded that *M. toringo* was phylogenetically close to *M. angustifolia.*

## Introduction

*Malus toringo* (Siebold) Siebold ex de Vriese is a worthy species in the genus Malus (family Rosaceae) mainly distributed in North America and Asia in a mixed forest or shrublands, with an elevation of 100–2000 m (Editorial Committee of Flora of North America 2015). In recent years, in addition to its ornamental value, the medicinal value of its fruits has also been developed to treat food stagnation diseases (Cui et al. 1993). However, its chloroplast genome has not been reported. Therefore, we examined the structure of complete chloroplast (CP) genome of *M. toringo* to explore the phylogenetic relationship in genus Malus by comparing it with other species’ chloroplast genome sequences within this genus.

The voucher specimen (BOP040556) of *M. toringo* was preserved in the herbarium, Institute of Botany, Chinese Academy of Sciences (PE). Molecular material was collected from the Royal Botanic Gardens, Kew (England; N 51° 28′ 43.57″, W 0° 17′ 44.12″). Total genomic DNA was isolated via modified cetyltrimethylammonium bromide (mCTAB) method (Li et al. 2013). An Illumina DNA library was constructed using total DNA and the DNA library was sequenced on Illumina HiSeq2500 platform for paired-end 150 bp reads (PE150). The genome was assembled de novo using SPAdes 3.9 (Bankevich et al. 2012) and annotated on Plann with referenced to *M. angustifolia* (NC045410). Finally the complete chloroplast genome was corrected with Sequin (Huang and Cronk 2015) and submitted to GenBank under accession number MT593044.

The complete chloroplast genome of *M. toringo* is 160,039 bp in length with a typical quadripartite structure. It includes two copies of inverted repeats (IRs, 26,357 bp), a large single copy (LSC, 88,142 bp), and a small single copy (SSC, 19,183 bp). Overall GC content was 36.6% and those in LSC, SSC, and IR regions were 34.2%, 30.4%, and 42.7%, respectively. A total of 128 genes were annotated, containing 83 protein-coding genes (PCGs), 37 transfer RNA genes (tRNA), and eight ribosomal RNA genes (rRNA).

**Figure 1.**
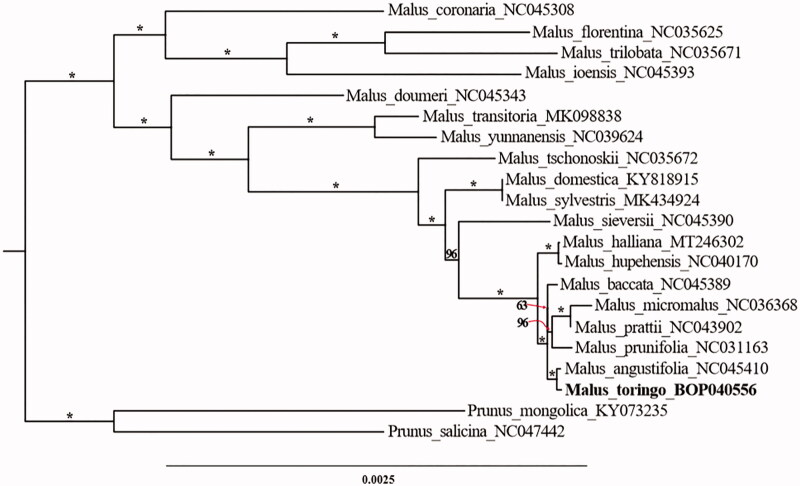
Phylogenetic tree based on plastid genomes using the ML method. Ultrafast bootstrap（UFBoot）values are shown above the nodes, with 1000 bootstrap replicates. * represents that this result is 100% supported.

To resolve the phylogenetic relationships, the complete chloroplast genome sequences of eighteen species from genus Malus and two species from genus Prunus as outgroups were downloaded from the GenBank. Multiple sequences alignment was executed by MAFFT (Katoh and Standley 2013). The maximum-likelihood (ML) phylogenetic tree with 1000 bootstrap replicates was constructed using the IQ-TREE with the best-fit model determined using ModelFinder (Nguyen et al. 2015; Kalyaanamoorthy et al. 2017). The phylogenetic tree indicated that *M. toringo* was phylogenetically close to *M. angustifolia* (100% ultrafast bootstrap support, Figure 1) (Hoang et al. 2018). Our findings provide valuable information on the chloroplast genome of the genus and related group in Rosaceae.

## Data Availability

The data that support the findings of this study are openly available in NCBI GenBank database at (https://www.ncbi.nlm.nih.gov) with the accession number is MT593044, which permits unrestricted use, distribution, and reproduction in any medium, provided the original work is properly cited.
